# Kringle-Dependent Inhibition of Plasmin-Mediated Fibrinolysis by Native and Citrullinated Core Histones

**DOI:** 10.3390/ijms26125799

**Published:** 2025-06-17

**Authors:** Erzsébet Komorowicz, Anna Gurabi, András Wacha, László Szabó, Olivér Ozohanics, Krasimir Kolev

**Affiliations:** 1 Department of Biochemistry, Semmelweis University, 1085 Budapest, Hungary; gurabi.anna@stud.semmelweis.hu (A.G.); szabo.laszlo@ttk.hu (L.S.); ozohanics.oliver@semmelweis.hu (O.O.); kolev.krasimir@semmelweis.hu (K.K.); 2 Institute of Materials and Environmental Chemistry, HUN-REN Research Centre for Natural Sciences, 1117 Budapest, Hungary; wacha.andras@ttk.hu

**Keywords:** neutrophil extracellular traps, thrombosis, fibrin

## Abstract

The fibrin matrix of thrombi is intertwined with neutrophil extracellular traps (NETs) containing histones that render resistance to fibrinolysis. During NET formation, histones are citrullinated. Our study addresses the question of whether citrullination modifies the fibrin-stabilizing effects of histones. We studied the structure and viscoelastic properties of fibrin formed in the presence of native or citrullinated H1 and core histones by scanning electron microscopy, clot permeation, and oscillation rheometry. The kinetics of fibrin formation and its dissolution were followed by turbidimetry and thromboelastometry. Co-polymerizing H1 with fibrin enhanced the mechanical strength of the clots, thickened the fibrin fibers, and enlarged the gel pores. In contrast, the addition of core histones resulted in a reduction in the fiber diameter, and the pores were only slightly larger, whereas the mechanical stability was not modified. Plasmin-mediated fibrinogen degradation was delayed by native and citrullinated core histones, but not by H1, and the action of des-kringle1-4-plasmin was not affected. Plasmin-mediated fibrinolysis was inhibited by native and citrullinated core histones, and this effect was moderated when the kringle domains of plasmin were blocked or deleted. These findings suggest that in NET-containing thrombi that are rich in core histones, alternative fibrinolytic enzymes lacking kringle domains are more efficient lytic agents than the classic plasmin-dependent fibrinolysis.

## 1. Introduction

Histones are basic proteins with arginine (Arg) and lysine (Lys) comprising 20–25% of their amino acid sequences. They contribute to the packaging of nuclear DNA into nucleosomes, in which DNA is wrapped around a core histone octamer consisting of the Arg-rich H2A, H2B, H3 and H4 [1]. The H1 linker histone stabilizes this structure by binding to the DNA strand at both entry and exit sites. While all histones have a short N-terminal tail prone to post-translational modifications, H1 also presents a long, C-terminal Lys-rich tail. Numerous post-translational modifications of histones have been described, which can modify histone–DNA interactions, and thereby regulate transcription [2]. Specific Arg residues in both core and linker histones have been identified as citrullination targets for the nuclear peptidylarginine deiminase 4 enzyme (PAD4), and the loss of Arg-related positive charges could contribute to chromatin decondensation [3,4,5,6]. PAD4-mediated citrullination of histones is a key, albeit, depending on the stimuli, not always an essential event in neutrophil extracellular trap (NET) formation [7,8]. The release of NETs is most probably a primordial innate immune reaction to invading pathogens: the sticky DNA meshwork helps to localize the invaders, while the other released components exert their anti-microbial functions. In addition to the benefits in infections, overworking NETs could potentially harm parenchymal organs and the vasculature, as detailed in recent reviews [9,10]. The plasma levels of citrullinated histones, as NET-markers were found to correlate with disease severity in autoimmune diseases, sepsis, or disseminated intravascular coagulopathy (DIC) [11,12,13,14]. NET- or, more specifically, histone-targeted countermeasures ameliorated, at least in part, the organ damage both in vitro and in animal models, which further supports the rationale behind the development of anti-histone therapy for human diseases [10,15,16].

NETs have been detected in human arterial and venous thrombi, ex vivo, and the capacity to form NETs is strongly related to thrombus formation in animal models [17,18,19]. We and others have characterized the prothrombotic and clot structure-modifying effects of histones and DNA, including their anti-fibrinolytic effects in tissue-type plasminogen activator (tPA)-mediated and plasmin-mediated fibrinolysis models [20,21,22,23,24]. However, histones may differentially modulate the pro-fibrinolytic effects of various heparin types; in fact, they might act in favor of fibrinolysis by enhancing single-chain u-PA activation into its two-chain form [25,26]. Moreover, in the presence of activated neutrophils, an alternative fibrinolytic system may also function, which involves neutrophil elastase that removes the N-terminal 4 kringle domains of plasminogen to form des-1-4-plasminogen (miniplasminogen), which, in turn, will be activated by tPA to miniplasmin [27]. Kringle domains contain lysine-binding sites and play a significant role in several steps of the regulation of fibrinolysis, including plasminogen activation, fibrin cleavage and interaction with plasma protease inhibitors [28,29]. The serine-protease domain of (mini)plasmin is a trypsin-homolog with substrate specificity for arginyl- and lysyl-peptide bonds. Thereby, it is not surprising that the basic histones are substrates of plasmin [24]. PAD4-mediated citrullination sites were found in all histone variants [3,4,5,6], but how this modification modulates the interplay between histones and fibrinolysis has not been investigated in detail.

In our current work, we aimed to compare the effects of the Lys-rich H1 linker histone and the Arg-rich core histones on fibrin clot structure and dissolution, as well as to assess the functional consequences of their PAD4-mediated citrullination in fibrinolysis. In addition, we also looked at the kringle dependency of the histone effects, applying a chemical blockade of the kringles in plasmin, a kringle-deleted plasmin variant (miniplasmin), and the kringle-less protease elastase. Miniplasmin and elastase are not only helpful means to explore the role of kringles, but their action is relevant to in vivo fibrinolysis in thrombi containing NETs, because neutrophil elastase is incorporated in the NET meshwork and, there, it can partially digest plasminogen to generate miniplasminogen.

## 2. Results

### 2.1. Characterization of Native and Citrullinated Histones by Mass Spectrometry

Two different commercially available preparations of calf thymus histones—designated as types III-S and VIII-S by the manufacturer—were applied in this study. Liquid Chromatography/Tandem Mass Spectrometry (LC-MS/MS) and sequence analysis of native and citrullinated histones after tryptic digestion confirmed that type III-S histones belonged mostly to the group of Lys-rich H1 variants, whereas type VIIIS was a mixture of Arg-rich core histones, mostly H4 and H2 variants, with H3 as a minor component (Figure S1 in the Supplementary Materials). Accordingly, in this paper, type III-S and VIII-S preparations will be referred to as H1 and core histones, respectively. A comparison of the LC-MS/MS chromatograms of the native vs. citrullinated histones supported the role of citrullination as the cause for the decreased amount of specific peptides, which appear to vanish from the native chromatogram and appear in the PAD4-treated samples (Figure S2). Sequence analysis of abundant peptides identified the specific Arg residues serving as citrullination sites in the various histone subtypes (listed in Table S1).

### 2.2. Modulation of the Fibrin Structure by Histones

The fibrin network structure formed in the presence of native or citrullinated histones was characterized with three parameters: fibrin fiber thickness assessed by scanning electron microscopy (SEM), porosity estimated from fluid permeability measurements, and turbidity, which reflects the fiber mass/length ratio dependent on fiber cross-sectional area and protofibril density in the fibers. These structural characteristics of the composite fibrin clots are summarized in Table 1. H1 histone co-polymerized with fibrin caused thickening of the fibers as we have previously described [22]. However, thinner fibrin fibers were observed in the presence of core histones. Both H1 and core histones increased the network porosity but to a different degree. The Darcy constant of the clots was more than doubled by H1, and increased by 25% by core histones, concurrent with a similar extent of turbidity increase (2.3- and 1.5-fold, respectively). All network-modulatory effects of histones were dampened by their PAD4-mediated citrullination.

### 2.3. Effects of Histones on the Biomechanical Properties of Fibrin

**Figure 1:**
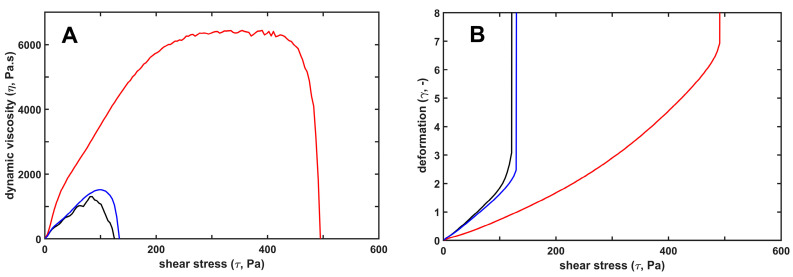
Mechanical stabilization of fibrin structure by H1, as opposed to core histones. H1 (red) or core (blue) histones at 25 mg/L were added to 7.4 µM fibrinogen and the mixture was clotted with 8 nM thrombin for 12 min under oscillatory shear in the measurement gap of the rheometer, as detailed in Materials and Methods. Thereafter, the clots were subjected to stepwise increasing shear stress, while the resulting strain was continuously monitored. Panel (**A**) shows the dynamic viscosity values calculated as the ratio of stress/strain. Panel (**B**) shows the relative deformation. Black lines depict the viscoelastic behavior of pure fibrin gel for comparison. The presented curves are representatives of a series of experiments (*n* = 4–8); numerical data and statistics are shown in Table 2.

Since fibrin formation and dissolution proceed under flow conditions in vivo, the biomechanical properties of the clots may significantly influence both fibrin deposition and lysis. We have previously described the clot-strengthening effects of H1 histone: viscoelastic measurements by oscillation rheometry showed higher storage and loss moduli, and higher critical shear stress needed for clot disintegration [22]. In the current study, we confirmed these H1 effects and compared them to the core histone effects (Figure 1). Fibrin clots polymerized in the presence of core histones were mechanically indistinguishable from pure fibrin clots, while citrullination of H1 histone partially reversed its clot-strengthening effects (Figure 1, Table 2). Interestingly, two parameters, the loss tangent (reflecting how elastic the substance is, i.e., to what extent the imposed deformation is reversible) and the higher maximal bearable deformation with H1, were not hampered by its citrullination, whereas storage and loss moduli, as well as critical shear stress needed for clot disintegration, decreased.

### 2.4. Kinetics of Fibrin Formation and Resolution in the Presence of Histones

**Figure 2:**
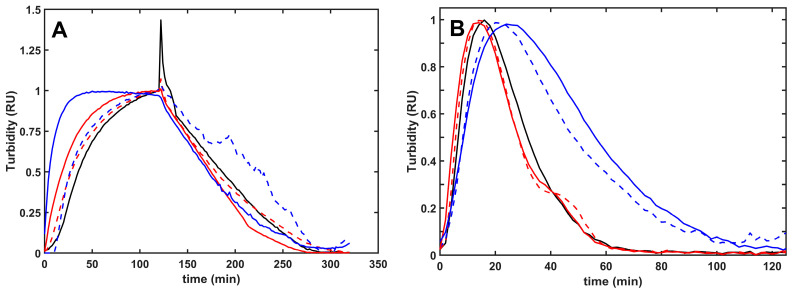
Plasmin-mediated fibrinolysis of composite fibrin/histone clots in extrinsic and intrinsic models. (**A**): Extrinsic lysis model: fibrinogen (6 µM) was clotted with 2 nM thrombin in the absence (black) or presence of 100 mg/L native (solid line) or citrullinated (dashed line) H1 (red) or core (blue) histones, and after a 2 h clotting period, 500 nM plasmin was layered on the clot surface to initiate fibrin dissolution. (**B**): Intrinsic lysis model: fibrinogen (6 µM) containing none (black) or 75 mg/L native (solid line) or citrullinated (dashed line) H1 (red) or core (blue) histones was clotted with 10 nM thrombin with 8 nM plasmin added at the same time to induce fibrinolysis simultaneously with clotting. The acending and descending parts of the turbidimetric curve recorded as absorbance at 340 nm wavelength indicate fibrin formation and dissolution. The presented kinetic curves are averages from 5 parallel measurements on the same day and were normalized considering maximal turbidity as 1.

Composite fibrin/histone clots developed faster (shorter clotting times) and lytic resilience correlated with the fibrin structure. The more porous network of thicker fibers formed in the presence of H1 was dissolved faster than pure fibrin, whereas lysis time of the fibrin/core histone structures was not significantly changed (Figure 2, Table 3). Intrinsic fibrinolysis with incorporated plasmin, however, was significantly delayed by core histones, the effect of which was moderated by citrullination (Figure 2, Table 3).

In addition to the static turbidimetric assays, we also applied the ClotPro thromboelastometer, in which the samples are formed and lysed under superimposed oscillatory stress and signal detection is based on dynamic interactions of the fibrin fibers formed in the gap between the oscillating measurement cup wall and the stationary pin. Following fibrin formation and lysis initiated with incorporated tPA and plasminogen, core histones exerted the most pronounced effects. Native core histones delayed both fibrin formation and lysis, and deamination of its arginine residues reduced this effect (Table 4). The effects of H1 were moderate or non-significant both in the static and the dynamic setups. The oscillatory stress in the thromboelastometric assay dampened the relative core histone effects on both fibrin formation and resolution; the prolongation of clotting time was reduced from 1.53-fold to 1.21, while that of lysis time from 4.45-fold to 1.78 (Table 4).

### 2.5. The Role of Kringle Domains in the Interplay of Histones and Plasmin in the Resolution of Fibrin Clots

**Figure 3:**
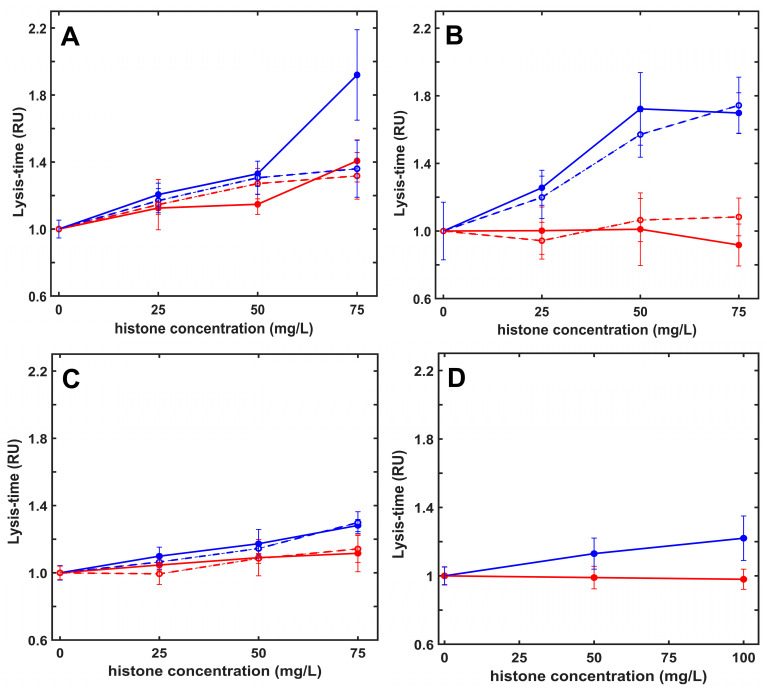
Histones inhibit plasmin-mediated fibrinolysis in a kringle-dependent manner. Fibrinogen (6 µM) containing native (solid line) or citrullinated (dashed line) H1 (red) or H3 (blue) histones at indicated concentrations was clotted with 10 nM thrombin in the presence of 8 nM plasmin (panel (**A**)), kringle-blocked plasmin (panel (**B**)) or miniplasmin (panel (**C**)), or 26 nM elastase (panel (**D**)) added at the same time to induce fibrinolysis simultaneously with clotting. Lysis time (LT50) was computed on the descending part of the recorded turbidimetric curve in a similar setup, as illustrated in Figure 2B, and plotted in relative units (RU) considering LT50 in the absence of histones as a reference (43 min, 78 min, 98 min and 37 min for plasmin, kringle-blocked plasmin, miniplasmin, and elastase, respectively). Data are plotted as mean ± SD values (*n* = 10–15).

To assess the role of the five kringle domains (K1–5) of plasmin, alternative enzymes were tested: (i) plasmin pre-treated with 6-aminohexanoate to block the high-affinity Lys-binding site located on the K1–3 domains, (ii) miniplasmin lacking the first four kringles, and (iii) elastase, a serine protease lacking kringle domains and fibrin specificity. The lysis time prolongation in the presence of native or citrullinated histones showed a significant kringle-dependence. The approximately 30% H1-mediated inhibition of fibrinolysis was seen only with native plasmin, whereas core histones caused 100%, 70% and 28% LT50 prolongation with plasmin, K1–3-blocked plasmin, and miniplasmin, respectively. When elastase was applied at concentrations producing fibrinolytic activity equivalent to the action of native plasmin in pure fibrin, native core histones prolonged its lysis time by 33%, corresponding to the histone sensitivity of miniplasmin (Figure 3).

### 2.6. Effects of Histones on the Fibrinogen Susceptibility to Plasmin Digestion

**Figure 4:**
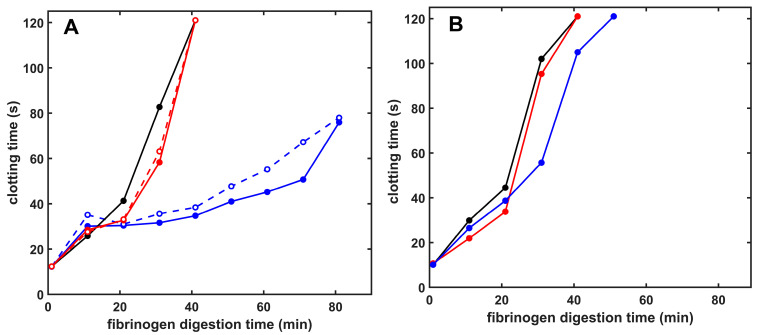
Plasmin- and miniplasmin-mediated loss of fibrinogen clottability in the presence of native or citrullinated histones. Fibrinogen (6 µM) was incubated with 12 nM plasmin (**A**) or miniplasmin (**B**), and thrombin at concentration set to yield a 10 s clotting time with undegraded fibrinogen was added to samples withdrawn after various times to assess the presence of residual clottable fibrinogen. Samples with clotting times longer than 120 s were plotted as 121 s and considered non-clottable due to extensive fibrinogen degradation. Additives in the panels are as follows: (**A**) Native (solid) or citrullinated (dashed) H1 (red) or core (blue) histones at 50 mg/L (**B**): Native H1 (red) or core (blue) histones at 50 mg/L concentrations; control curves are all black. Representative curves are presented, which were repeated on at least 3 different days to verify the observed tendencies.

Given the differential effects of the two tested histone types on fibrin dissolution, we turned to fibrinogen, another physiologically important plasmin substrate. Fibrinogen cleavage by plasmin results in loss of clottability, which we could follow by measuring the clotting time of the residual fibrinogen in samples digested for various time intervals with plasmin. Core histones protected fibrinogen against loss of clottability, whereas the H1 effects were very mild (Figure 4). Citrullination of histones did not alter their modulatory effects in this model (Figure 4). Miniplasmin-mediated fibrinogen degradation, however, was much less affected by core histones, whereas, similarly to plasmin, H1 did not significantly affect the fibrinogenolysis by miniplasmin (Figure 4).

**Figure 5:**
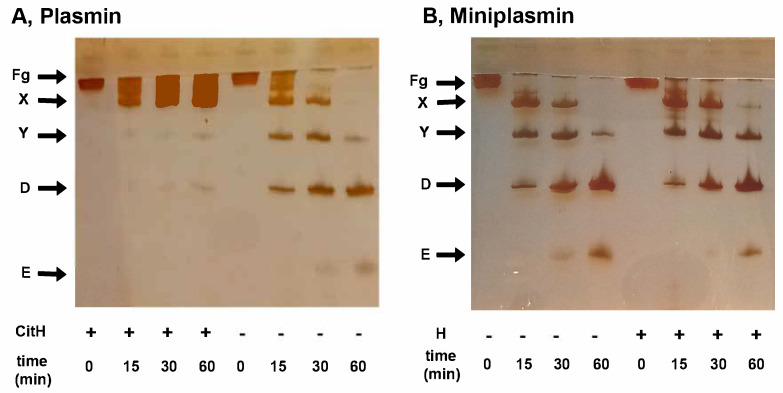
Formation of fibrinogen degradation products by plasmin or miniplasmin in the presence of histones. Fibrinogen (1.5 µM) was incubated with 12 nM plasmin (panel (**A**)) or miniplasmin (panel (**B**)) in the absence or presence of 50 mg/L native (**B**) or citrullinated (**A**) core histones. At time intervals, the samples were withdrawn, heated and subjected to SDS-PAGE in a 7.5% homogeneous gel, followed by silver staining. Band labels: Fg, fibrinogen; X/Y/D/E, fibrinogen degradation products.

Visualization of the fibrinogen degradation products by SDS-PAGE verified the native or citrullinated core histone-mediated delay in the plasmin-mediated formation of the non-clottable fibrinogen degradation products Y and D, while miniplasmin was insensitive to the core histones (Figure 5).

### 2.7. Effects of Histones on Plasma Clot Formation and Lysis

Lastly, we evaluated how the findings on the kinetics of pure fibrin clot formation and lysis would translate into the more complex plasma environment (Table 5). H1 prolonged, whereas core histones shortened the clotting time of plasma clots, and these effects were not significantly modified by the PAD4 pretreatment of the histones. The lysis time of the pre-formed clots with tPA was prolonged by 14–30% when histones were co-polymerized into the plasma clots, and histone citrullination caused only minor changes in the lytic resilience of the clots (Table 5). Because extracellular histones are commonly found in the structure of neutrophil extracellular traps, where their interaction with DNA may be driven by Arg-related positive charges, we tested the combined effects of histones and DNA on the kinetics of plasma clot formation and lysis. In the clotting phase, core histones did not modulate the clotting time-shortening effect of DNA, but H1 did. The combined effect of native H1+DNA on clotting was indistinguishable from the effect of H1 alone. Citrullination of H1 modulated the acceleration of clotting by the H1+DNA complex, whereas citrullination of core histones rendered their complex with DNA an even stronger accelerator of plasma clotting than DNA alone. The presence of histones—either native or citrullinated—caused only minor (if any) changes in the inhibitory effects of pure DNA on clot lysis.

## 3. Discussion

Since the discovery of NETs in the cross-section of inflammation and thrombosis, an increasing number of pathologies have been associated with NETs, and, in many cases, disease severity has been linked to elevated plasma levels of NET-components like histones or DNA [11,12,13,14]. While both linker and core histones have been reported as targets of NET-related citrullination, core histones including H3 have drawn more attention than linker histones, and citrullinated H3 has been proposed as a biomarker of prognostic value for NET-related thrombus formation [6,13]. Neutrophil infiltration and NET formation impede therapeutic thrombolysis in stroke patients, which led to anti-NET approaches including DNase I in clinical trial as an adjuvant in tPA-mediated thrombolysis. Histone-targeted approaches are also being developed, but while much of the focus is on their cytotoxic and prothrombotic effects, potential fibrinolysis modulation remains in the shade [30,31,32,33]. Development of both diagnostic and therapeutic tools may benefit from the differential characterization of histone types in terms of their various cytotoxic, and prothrombotic and antifibrinolytic effects, as well as from the citrullination-mediated modulation of these histone effects.

The cytotoxic effects of histones, as polycationic proteins, have been attributed to their interaction with negatively charged phospholipid head groups in the membrane, and H3 or H4 histones were found to be more detrimental to endothelial cells and neutrophils than H1 [33,34]. H4 and H3 were also the most efficient types that promoted platelet-dependent thrombin generation and platelet activation, while they inhibited protein C activation [20,21]. On the other hand, among the histone types, H1 was the most potent in neutralizing the pro-fibrinolytic effects of UFH and LMWH [25]. The hypercitrullinated H1 could serve as an autoantigen in systemic lupus erythematosus and Sjögren’s syndrome, and H1 was also found to increase astrocytic reactivity against neurons in neuro-inflammatory conditions [6,10]. The functional consequence of histone citrullination may vary, and, while citrullinated histones were less cytotoxic to endothelial cells, neutrophils, and bacteria, and proposed as curtailing immune reactions [33,34,35], hypercitrullinated autoantigens could drive the pathology in autoimmune diseases, and inhibition of citrullination by PAD4 inhibitors was protective against parenchymal organ damage [8,10,36]. Both native and citrullinated histones were visualized in ex vivo thrombi, and multiple interactions between native histones and the hemostatic–fibrinolytic system have been recognized, but how citrullination may modulate these interactions needs further clarification [16,17,18,19,23].

In our current work, we have characterized the differential effects of H1, the Lys-rich linker histone-type, and the Arg-rich core histones on fibrin formation and mechanical and lytic properties of the composite histone/fibrin clots. Histones showed dose-dependent effects in the 0–100 mg/L concentration range, which conforms to previous studies in the field [20,21,22,23,24,25,26], and we selected a concentration, depending on the histone sensitivity of the assay, at which native histone effects were pronounced and, hence, their potential dampening by citrullination could be determined. Circulating plasma levels of histones up to 140 mg/L have been published for critically ill patients, such as sepsis, trauma, or pancreatitis, and patient sera with more than 30 mg/L were cytotoxic to endothelial cells [37,38]. Citrullinated H3 levels have been proposed as biomarkers correlating with severity in stroke, cancer, and trauma, even though multiple technical issues may compromise its quantitative determination, including rapid clearance from plasma, antibody specificity, and stability of the standards [39]. Compared to systemic circulating histone levels, it would be more difficult to determine the local concentration of histones where histones might influence the hemostatic balance. The intense histone staining of neutrophil-rich thrombi suggests the focal accumulation of the NET components at sites of NET-related thrombus formation [22]. From data about another NET component, the human neutrophilic elastase the local concentration of histones at the site of their release can be estimated indirectly. While the elastase concentration in azurophilic granules is 160 g/L, it is measured in the order of 30 mg/L in the immediate pericellular zone of activated neutrophils and 0.02 or 0.2 mg/L in circulating blood of healthy individuals and cirrhotic patients, respectively, which suggests a 100–1000-fold dilution factor to be considered [40,41].

We have previously described that native H1 enhanced fibrin formation from purified fibrinogen and thrombin, and its incorporation into fibrin fibers resulted in a mechanically more stable network of thicker fibers enclosing larger pores [22,42]. Our current data show that citrullination modifies the histone-mediated effects on fibrin network characteristics, but even citrullinated H1 can significantly stabilize the fibrin structure, as reflected in the higher viscoelastic moduli, higher maximal bearable deformation, and the need for a higher critical shear stress to disintegrate the clots (Table 2). Core histones, however, induce slightly thinner fibers and bigger pores with a concurrent increase in network turbidity reflecting an increased fiber mass/length ratio, which depends on both fiber thickness and protofibril density in the fibers (Table 1). Such parameters may be consonant with less branched and thinner but more densely packed fibers in the core histone/fibrin network. Both fiber thickness and pore size are known to affect the mechanical stability of the clots. At an identical fibrin monomer concentration, thicker fibers with more protofibrils/fiber cross-area reinforce the mechanical stability of the matrix, but the necessarily accompanying reduced branching density compromises the mechanical resilience [43]. The fact that the core histone/fibrin, despite the thinner fibers and larger pores (Table 1), is not weaker than pure fibrin (Figure 1) can also be explained by the increased protofibril density in the thinner fibers, which altogether can maintain mechanical stability similar to that of pure fibrin.

The histone-mediated modulation of fibrinolysis with plasmin depended on the model system applied. Preformed fibrin dissolution with surface-applied plasmin was not significantly inhibited in the presence of histones, probably because of the more porous clot structure enabling enhanced plasmin penetration (Table 1 and Table 3). On the contrary, when plasmin was incorporated into the clots, native or citrullinated core histones caused an up to 2.4-fold or 1.5-fold prolongation of lysis time, and this effect was kringle-dependent, since kringle-blocked plasmin, miniplasmin lacking four kringles, or elastase devoid of kringles were much less inhibited (Figure 3). A similar, kringle-dependent inhibition by core histones was found when fibrinogen cleavage by plasmin or miniplasmin was monitored with two independent methods: the fibrinogen clottability assay and SDS-PAGE (Figure 4 and Figure 5). Core histones delayed the loss of clottability when fibrinogen was cleaved by plasmin, but miniplasmin was not affected. Citrullination of Arg residues mildly hampered the inhibition of plasmin-mediated fibrinolysis but did not modulate the effects of core histones on miniplasmin-mediated fibrinolysis nor fibrinogen cleavage (Figure 3, Figure 4 and Figure 5). One possible explanation for the kringle-dependent fibrinolysis inhibition by histones could be the long-appreciated perfect design of plasmin, sterically matching the kringle-dependent binding sites and the cleavage-susceptible peptide bonds in the fibrin protofibrils, rendering plasmin with high processivity and catalytic efficiency [28,29,44]. The histone–fibrin interaction may shield such critical plasmin-binding sites, or histone-mediated changes in fibrin fiber structure could result in loss of the kringle-dependent steric efficiency of plasmin, while miniplasmin and elastase are less influenced. Another explanation might be that histones, as alternative substrates for plasmin, compete with fibrin for plasmin, which could result in a dose-dependent inhibition of its fibrinolytic activity. Loss of Arg residues in citrullinated histones may decrease the affinity of plasmin for histones and relieve their competitive inhibitory effect on plasmin-mediated fibrinolysis, as we observed (Table 3 and Table 4). In this case, the kringle dependency of fibrinolysis inhibition might be related to a kringle-dependent recognition of histones as plasmin substrates. Nevertheless, citrullination of core histones does not relieve its inhibitory effect completely. The fact that the various histones exerted similar effects on plasmin-mediated fibrinogen and fibrin degradation, as well as on tPA-mediated fibrinolysis, which also includes the step of plasminogen activation to plasmin, points to plasmin activity as the primary target of histones in fibrinolysis modulation (Table 4). A comparison of fibrin dissolution under static and dynamic conditions did not bring a major switch in histone-mediated effects but clearly demonstrated that dynamic forces hampered the development of the fibrin structure and improved its dissolution: clotting time values for pure fibrin applying the same thrombin concentrations under static were 10-fold shorter than those under dynamic conditions, whereas a 4-fold lower concentration of tPA was sufficient to lyse fibrin under oscillating shear forces than under static conditions (reference values in Table 4).

The differential histone effects were also detectable in a more complex plasma environment. Thrombin-mediated formation of plasma clots was slowed down by H1 but accelerated in the presence of co-polymerized core histones and DNA, alone or in combination. Loss of some Arg-residues upon histone citrullination altered some histone effects, most profoundly the H1- and H1/DNA-mediated prolongation of clotting time, but dissolution of the plasma clots containing NET components was suppressed independently of histone citrullination (Table 5).

## 4. Conclusions

In summary, histones can differentially modulate the formation and network properties of fibrin clots. While H1 strengthens clot structure, core histones impede plasmin-mediated cleavage of both fibrinogen and fibrin in a kringle-dependent manner. Citrullination dampens some histone effects, but even citrullinated histones can mechanically stabilize and prolong the lifetime of fibrin clots. Our current findings contribute to an improved understanding of the in vivo consequences of the formation of fibrin(ogen)–histone complexes, and the co-localization of histones with fibrin in plasma clots of cancer patients reported previously [22,24,34,45]. Moreover, an elevated histone content of clots retrieved from stroke patients has been shown to correlate with unsuccessful recanalization upon tPA-mediated thrombolytic therapy [46]. Our work supports the therapeutic potential of anti-histone pan-specific strategy in thrombotic disorders, similarly to the recently presented anti-histone antibody recognizing both H2 and H4 in autoimmune diseases [47]. The kringle dependence of histone-mediated inhibition of fibrino(geno)lysis by plasmin substantiates the role of alternative fibrinolytic enzymes that are less hindered in NET-rich clots, such as elastase or miniplasmin. Indeed, elastase-specific digests of cross-linked fibrin have been observed at elevated levels in sepsis, malignancy or disseminated intravascular coagulopathy, which supports the concept that alternative enzymes might contribute to clot dissolution, especially if the classic tPA–plasminogen system is compromised by inflammation and NETs [48,49,50].

## 5. Materials and Methods

Human fibrinogen (plasminogen free), histones from calf thymus of type IIIS containing predominantly H1 histone (H1) and type VIIIS containing predominantly arginine-rich core histones, and calf thymus DNA were purchased from Merck KGaA (Darmstadt, Germany), dissolved in Hepes-buffered saline (HBS; 10 mM Hepes 150 mM NaCl pH 7.4), and stored at −20 °C until use. Peptidyl Arginine Deiminase Type 4 (PAD4) was also from Merck. The chromogenic substrate for plasmin Spectrozyme-PL (H-D-norleucyl-hexahydrotyrosyl-lysine-p-nitroanilide) was from BioMedica Diagnostics (Windsor, NS, Canada). Bovine thrombin and porcine pancreatic elastase were purchased from Serva (Heidelberg, Germany). Thrombin was further purified by ion-exchange chromatography on sulfopropyl-Sephadex yielding a preparation with specific activity of 2100 IU/mg [51], and 1 IU/mL was considered equivalent to approximately 10.7 nM by active site titration [52]. Recombinant tPA was from Boehringer Ingelheim, Ingelheim am Rhein, Germany. Isolation of plasminogen from normal plasma, preparation of its des-kringle1-4 derivative, miniplasminogen, and their activation and measurement of active (mini)plasmin concentration were carried out, as previously described in our laboratory [53].

### 5.1. Preparation and Characterization of Citrullinated Histones

Histones at a 1 g/L concentration were treated with the PAD4 enzyme in HBS containing 10 mM CaCl_2_ and 1 mM dithiothreitol and the degree of citrullination was monitored in an assay based on the accelerating effect of native histones on the fibrinogen clotting by thrombin and the loss of this effect upon citrullination as previously described [34]. In this assay, 1 U/mL PAD4 citrullinated completely 1 g/L histone within 2 h at 37 °C. Freshly citrullinated histones were immediately diluted further according to the experimental protocol. In all cases, the stock of citrullinated histone was diluted at least 10-fold to achieve dilutions of DTT and PAD4 that do not interfere with fibrinogen clotting and fibrinolysis.

Native and citrullinated histone samples were also analyzed by liquid chromatography coupled mass spectrometry to characterize the composition of type III-S and VIII-S, verify their citrullination and identify citrullination sites. Detailed methods and data are described in the Supplementary Materials. Based on the composition data, type III-S and VIII-S are referred to as H1 and core histones, respectively.

### 5.2. Characterization of the Structure of Fibrin Clots

Scanning electron microscopy (SEM) was used for the measurement of fiber thickness in fibrin. Fibrin clots were prepared in duplicates: (i) 7.4 μM fibrinogen in 10 mM HEPES buffer pH7.4 containing 150 mM NaCl (HBS); (ii) 1 g/L histone in HBS containing 10 mM CaCl_2_ and 1 mM dithiothreitol, and (iii) 50 nM thrombin in HBS were combined in a 8:1:1 volume ratio in Eppendorf tubes and incubated at 37 °C for 2 h to allow for clot formation (parallel samples were clotted in microtiter plate wells to monitor the course of clotting with turbidimetry at 340 nm wavelength, and the plateauing of the turbidimetric values was the criterion for completion of clotting). Clots were thereafter processed for SEM imaging as detailed previously [54], and images were taken with a scanning electron microscope EVO40 (Carl Zeiss GmbH, Oberkochen, Germany). SEM images were analyzed to determine the distribution of fibrin fiber diameters using self-designed program functions running under the Image Processing Toolbox v.11.7 of MatlabR2023a (The Mathworks, Natick, MA, USA), as previously described [55,56]. The diameter of 300 fibers was measured on 4–6 SEM images. Clots were characterized by the median and low-bottom quartile of the diameter values.

Fluid permeability assays were performed according to our previously described method to characterize clot porosity [57]. Fibrinogen at 8.8 µM in HBS with or without 100 mg/L citrullinated or non-citrullinated histones was added to 15 nM thrombin, and after brief mixing, the 150 µL mixture was allowed to clot at the bottom of 1000 µL pipette tips. The tips were removed from the pipette after 90 s and placed in a humid chamber for 90 min at 37 °C to reach complete clotting. Thereafter, a 40 cm long silicon tubing was attached to the bottom of the clots in a vertical position and a 17 cm high liquid pressure head was created by carefully filling up the tip and assembling one more 5 mL tip on top of it. After 10 min of washing with HBS, volumetric rate of permeated buffer was calculated based on the inner diameter of the tubing and the mm length of the throughput liquid read every 10 min for at least 2 h. The fluid pressure head was kept constant by refilling the buffer reservoir with HBS. Pore size of the fibrin clots was estimated on the basis of the permeation coefficient (Darcy’s constant, Ks):

$$K_{s}=\frac{Q\times n\times L}{t\times A\times ∆P}$$

where *Q* = volume of permeated buffer (cm^3^); *n* = viscosity of buffer (10^−2^ poise = 10^−7^ N.s/cm^2^); *L*= length of clot (1.5 cm); *t* = time (s); *A* = cross-sectional area of clot (0.09 cm^2^); Δ*P* = Pressure drop (0.170 N/cm^2^). Each experiment was carried out in 3–4 replicas, repeated at least 3 times on different days.

Viscoelastic properties of the fibrin clots were studied with oscillation rheometry, as previously described [54]. Fibrinogen (7.4 µM in HBS) was pre-mixed with 50 mg/L citrullinated or non-citrullinated histones and clotting was initiated with 8 nM thrombin. The clotting mixture was transferred to the stationary plate of HAAKE RheoStress1 oscillation rheometer (Thermo Scientific, Karlsruhe, Germany), the cone (Titanium, 2° angle, 35 mm diameter) of the rheometer was brought to the gap position, and an oscillatory shear strain (**γ**) of 0.015 at 1 Hz was imposed at 2 min after clot initiation. Storage (G′) and loss (G″) moduli were measured for 10 min with HAAKE RheoWin data manager software v.3.50.0012 (Thermo Scientific) to monitor the clot formation. Thereafter, the flow limit of the fibrin gels was measured in the same samples, increasing the applied shear stress (τ) from 0.01 to 1000 Pa stepwise in 300 s, and the resulting strain was used for calculation of the dynamic viscosity (η). The gel–fluid transition in the fibrin structure was indicated by an apparent fall in viscosity, the point of which could be characterized by two parameters: the maximal bearable strain (γ_max_) preceding the abrupt fall in viscosity and the critical shear stress τ_0_ that resulted in γ_max_.

### 5.3. Fibrin(ogen)olytic Assays

Cleavage of fibrinogen with plasmin or miniplasmin in the presence of native or citrullinated histones was monitored by measuring the clotting time of residual clottable fibrinogen in the incubation mixture. Fibrinogen at 2 g/L in 10 mM imidazole 150 mM NaCl pH 7.4 containing 2.5 mM CaCl_2_ was incubated with 12 nM (mini)plasmin in the presence of 0–25–50–100 mg/L histones at 37 °C, and at various times, 200 µL samples were withdrawn and clotted with 100 µL thrombin in a coagulometer KC-1A (Amelung, Lemgo, Germany). Thrombin concentration was set to yield a 10 s clotting time with undegraded fibrinogen. The curves presented show the clotting time values as a function of the time of (mini)plasmin digestion; clotting times longer than 120 s are plotted as 121 s, and such samples were considered non-clottable due to substantial fibrinogen degradation. Each set of experiments was repeated at least 3 times on different days to verify the reproducibility of the effects. The cleavage pattern of fibrinogen in similar experiments was also analyzed with SDS-PAGE in 7.5% homogenous gels, and the protein bands were visualized by silver staining, as previously described [29].

Fibrin formation and lysis were followed by recording light absorbance at 340 nm reflecting clot turbidity at 37 °C with a CLARIOstar spectrophotometer (BMG Labtech, Ortenberg, Germany) in the experimental settings below.

#### 5.3.1. Fibrinolysis with Plasmin and Miniplasmin Incorporated in Fibrin

Formation and lysis of composite fibrin clots was initiated by adding 140 μL fibrinogen (5 μM) in HBS containing histones, or an equal volume of citrullination buffer at various concentrations in the 0–200 mg/L range to 5 μL 300 nM thrombin and 5 μL 240 nM plasmin or miniplasmin in the wells of 96-well microtiter plates. In some experiments, plasmin was pre-mixed with 6-aminohexanoate at 62 µM to block Lys-binding sites located in the kringle domains of plasmin. The concentrations of the lytic and clotting agents were chosen to avoid incomplete clotting due to premature fibrinogen degradation. The maximal turbidity values were within a ±10% range of A_max_ values reached without the lytic agents.

#### 5.3.2. Fibrinolysis with tPA Incorporated in Fibrin

In order to assess the combined modulatory effects on both the generation and the fibrinolytic activity of plasmin, the above protocol was further modified: 10 nM plasminogen was included in the fibrinogen mixture, and for the initiation of fibrinolysis, 5 μL of 70 nM tPA was used. All other experimental details were the same, as described above for incorporated plasmin.

#### 5.3.3. Extrinsic Fibrinolysis with Plasmin Applied on the Surface of the Clots

Clots were formed in the microplate wells by adding 100 μL fibrinogen (6 μM) to 25 μL thrombin (10 nM) pre-mixed with histones or citrullination buffer. After 2 h of clotting (by which time clot turbidity always reached a plateau), 90 μL of plasmin (500 nM) was layered on the clot surface to initiate fibrinolysis.

#### 5.3.4. Formation and Extrinsic Lysis of Plasma Clots

Citrated normal plasma was supplemented with 0.6 μM plasminogen, recalcified with an equal volume of 25 mM CaCl_2_ and clotted with 9 nM thrombin in the presence of 0.1 g/L histones alone, or in combination with 0.09 g/L DNA. After 60 min, when all turbidity curves plateaued, 50 nM tPA was applied on the top of the clots and lysis was followed by turbidimetry.

For quantitation of the turbidimetric fibrinolytic experiments, clotting time (CT_90_ or CT_50_) was defined as the time needed to reach 90% or 50% of the maximal turbidity, A_max_, on the ascending part of turbidimetric curves. Lysis time was defined as the time elapsed from time zero (intrinsic lysis) or from the time of plasmin/tPA addition (extrinsic lysis) until the turbidity of the clot was reduced to half (LT_50_) of its maximal value on the descending part of the turbidimetric curves.

### 5.4. Statistical Analysis

The distribution of the data of fiber diameter was analyzed according to an algorithm used previously [55,56]: theoretical distributions were fitted to the empirical data sets and compared using Kuiper’s test and Monte Carlo simulation procedures. The statistical evaluation of other experimental measurements in this report was performed with the two-sample Kolmogorov–Smirnov test (Statistics and Machine Learning Toolbox v.12.5 of MatlabR2023a).

## Figures and Tables

**Table 1:** Effect of citrullination on the structural properties of the fibrin networks in the presence of H1 or core histones. Fibrin clots containing native or citrullinated histone H1 or core histone (at 100 mg/L) were examined for fibrin fiber diameter (scanning electron microscopy, median [interquartile range], network porosity (fluid permeability, K_s_ Darcy constant, mean (SD)), and fiber mass/length ratio (maximal turbidity, A_max_, mean (SD)) expressed in relative units compared to A_max_ of pure fibrin). Statistical significance at *p* < 0.05 according to Kuiper’s test following Monte Carlo simulation (fiber diameter) or two-sample Kolmogorov–Smirnov test (K_s_ and turbidity) is indicated by * in comparison to pure fibrin, or by ^#^ compared to the non-citrullinated counterpart of the same histone, *n* = 12–20.

	None	H1 Linker Histone	Core Histone	Citrullinated H1	Citrullinated Core
fiber diameter, nm	95.1 [44.6]	109.1 * [62.4]	72.1 * [34.5]	91.0 *^#^ [49.1]	77.8 *^#^ [42.7]
K_s_ 10^−9^ cm^2^	0.29 (0.06)	0.62 * (0.17)	0.36 * (0.08)	0.43 *^#^ (0.17)	0.29 ^#^ (0.05)
maximal turbidity	1.00 (0.06)	2.32 * (0.17)	1.55 * (0.25)	1.42 *^#^ (0.30)	0.92 *^#^ (0.18)

**Table 2:** Viscoelastic parameters of fibrin clots containing native or citrullinated histones. Fibrin clots containing native H1 or core histones, or 2-h citrullinated H1 histones (50 mg/L) were examined by oscillation rheometry, as detailed in Materials and Methods. The plateau values of the storage modulus (G′), loss modulus (G″) and loss tangent (G″/G′) at the end of the 12 min clotting phase, as well as the critical shear stress (τ_0_) at the maximal bearable relative deformation (γ_max_) before the gel/fluid transition in the fibrin structure, are presented as mean (SD). Asterisks (*) and pound signs (^#^) indicate *p* < 0.05 according to two-sample Kolmogorov–Smirnov test in comparison to pure fibrin, or testing the modulatory effect of citrullination, respectively, *n* = 4–8.

	G′ (Pa)	G″ (Pa)	G″/G′ (-)	γ_max_ (-)	τ_0_ (Pa)
no additive	34.6 (6.7)	2.8 (0.4)	0.082 (0.004)	1.9 (0.2)	100.4 (7.8)
native H1	82.1 * (10.6)	8.3 * (1.1)	0.101(0.002)	5.5 * (0.6)	678.5 * (89.4)
citrullinated H1	56.3 *^#^ (10.5)	5.5 *^#^ (1.1)	0.097 (0.010)	5.7 * (0.3)	412.8 *^#^ (64.1)
native core	36.2 (4.1)	2.9 (0.4)	0.087 (0.002)	2.1 (0.2)	120.8 (25.8)

**Table 3:** Formation of fibrin clots containing native or citrullinated histones and their lysis with plasmin in extrinsic and intrinsic setups. Clotting (CT90) and lysis time (LT50) values computed from measurements illustrated in Figure 2. Data are presented as mean (SD) in relative units compared to the respective CT90 and LT50 values for pure fibrin (83.7 min and 69.1 min with surface-applied plasmin, or 11.7 min and 38.6 min with incorporated plasmin, respectively). Statistical significance at *p* < 0.05 according to two-sample Kolmogorov–Smirnov test is indicated by * in comparison to pure fibrin, or by ^#^ compared to the non-citrullinated histone of the same type, *n* = 10–15.

	None	H1	Core	Citrullinated H1	Citrullinated Core
**Surface-applied plasmin**					
Clotting time	1.00 (0.04)	0.79 * (0.11)	0.62 * (0.29)	0.92 *^#^ (0.03)	0.84 *^#^ (0.09)
Lysis time	1.00 (0.13)	0.77 * (0.09)	0.93 (0.13)	0.85 *^#^ (0.15)	1.18 ^#^ (0.39)
**Incorporated plasmin**					
Clotting time	1.00 (0.10)	0.85 * (0.13)	1.33 * (0.22)	0.89 * (0.13)	1.26 * (0.23)
Lysis time	1.00 (0.12)	0.89 * (0.08)	2.40 * (0.73)	0.98 ^#^ (0.13)	1.49 *^#^ (0.22)

**Table 4:** Formation of fibrin clots containing native or citrullinated histones and their lysis with incorporated tPA and plasminogen. A, Turbidimetry. Fibrinogen (4.5 µM) containing 10 nM plasminogen and 75 mg/L native or citrullinated histone was clotted with 10 nM thrombin and 2 nM tPA was added at the same time to induce fibrinolysis simultaneously with clotting. Fibrin formation and dissolution were followed by turbidimetry at 340 nm wavelength, and clotting time (CT90) and lysis time (LT50) were computed on the ascending and descending parts of the individual curves, as detailed in Materials and Methods. B, Viscoelasticity. Fibrinogen (6.5 µM) containing 50 nM plasminogen and 100 mg/L native or citrullinated histone was clotted with 10 nM thrombin in the measurement cup of the ClotPro instrument and 0.5 nM tPA was added at the same time to induce fibrinolysis simultaneously with clotting. Clotting time and lysis time values were determined, as detailed in Materials and Methods. Data are presented as mean (SD), in relative units compared to pure fibrin. The reference clotting time values in pure fibrin were 10 and 128 s in the turbidity and the viscoelastic assay, respectively, whereas the corresponding reference lysis time values were 40 and 56 min. Statistical significance at *p* < 0.05 according to two-sample Kolmogorov–Smirnov test is indicated by * in comparison to pure fibrin, or by ^#^ compared to the non-citrullinated histone of the same type, *n* = 6–10.

	None	H1	Core	Citrullinated H1	Citrullinated Core
**A, turbidity**					
Clotting time	1.00 (0.08)	1.21 * (0.11)	1.53 * (0.31)	1.07 ^#^ (0.07)	1.25 *^#^ (0.19)
Lysis time	1.00 (0.11)	1.01 (0.23)	4.45 * (0.81)	1.15 (0.39)	2.08 *^#^ 0.031)
**B, viscoelasticity**					
Clotting time	1.00 (0.04)	1.02 (0.04)	1.21 * (0.01)	1.13 * (0.08)	1.06 ^#^ (0.14)
Lysis time	1.00 (0.08)	0.97 (0.04)	1.78 * (0.36)	0.96 (0.24)	1.20 *^#^ (0.27)

**Table 5:** Formation and tPA-mediated lysis of pre-formed plasma clots co-polymerized with histones and/or DNA. Plasma clots supplemented with 0.6 µM plasminogen and 0.1 g/L native or citrullinated H1 or core histones, alone or in combination with 0.09 g/L DNA, were pre-formed in microplate wells, and clot lysis was initiated with 50 nM tPA applied to the surface of the clots at 60 min, when all turbidimetric curves had reached their plateau values. Clotting and lysis times were defined, as described in Materials and Methods, and their mean (SD) values are presented in relative units compared to plasma clots without any additive. Statistical significance at *p* < 0.05 according to two-sample Kolmogorov–Smirnov test is indicated by * in comparison to pure plasma clots, by ^#^ in comparison to the non-citrullinated histone of the same type, and by ^φ^ in comparison to pure DNA, *n* = 10–20.

	H1	Core	DNA	H1 + DNA	Core + DNA
**Native histones**					
Clotting time	1.85 * (0.35)	0.77 * (0.32)	0.80 * (0.09)	1.79 *^φ^ (0.31)	0.72 * (0.19)
Lysis time	1.14 * (0.12)	1.28 * (0.11)	1.27 * (0.15)	1.14 *^φ^ (0.16)	1.21 * (0.15)
**Citrullinated histones**					
Clotting time	1.61 *^#^ (0.16)	0.76 * (0.21)	n.a.	1.21 *^#φ^ (0.09)	0.49 *^#φ^ (0.11)
Lysis time	1.24 *^#^ (0.17)	1.28 * (0.17)	n.a.	1.18 *^φ^ (0.16)	1.30 *^#^ (0.14)

## Data Availability

The original contributions presented in this study are included in this article and its Supplementary Materials. Further inquiries can be directed to the corresponding author.

## Supplementary Materials

The following supporting information can be downloaded at: https://www.mdpi.com/article/10.3390/ijms26125799/s1.

## Abbreviations

The following abbreviations are used in this manuscript:

tPA	Tissue plasminogen activator
NET	neutrophil extracellular trap
PAD4	Peptidyl Arginine Deiminase Type 4
SEM	Scanning Electron Microscopy
