# Supplementary material for: Kringle-Dependent Inhibition of Plasmin-Mediated Fibrinolysis by Native and Citrullinated Core Histones
Source: Int J Mol Sci. 2025 Jun 17;26(12):5799. doi: 10.3390/ijms26125799 (PMC12193439; doi:10.3390/ijms26125799)
Supplement: Supplementary file 1 [file ijms-26-05799-s001.zip › ijms-3597116-supplementary.pdf]

Kringle-Dependent Inhibition of Plasmin-Mediated Fibrinoly-sis by Native and Citrullinated Core Histones

Erzsébet Komorowicz, Anna Gurabi, András Wacha, László Szabó, Olivér Ozohanics, and Krasimir Kolev

Supplement

LC-MS and LC-MS/MS analysis of histone samples

Sample fractions of histone from calf thymus type III-S and type VIII-S were analyzed via LC-MS and LC-MS/MS to characterize their protein content.

LC-MS measurements were carried out on the unmodified samples using a Thermo Scientific Ultimate 3000 RSLC coupled to an LTQ-XL mass spectrometer. The sample components were separated on a 100x1 mm Hypersil GOLD aQ C18 column using mobile phases A – water with 0.15% formic acid and B – acetonitrile with 0.15% formic acid. The gradient elution started from 20% B kept for 2 minutes, increased to 85% B in 8 minutes and kept for 2 minutes. Flow rate was set to 0.1 mL/min and column temperature was set to 30°C. Data was recorded in positive mode in the 500-1600 m/z range.

Raw spectra were processed by baseline subtraction with a 40% threshold and MaxEnt 1 deconvolution was applied. The resulting protein masses can be seen below in Fig S1. The type III-S sample contained mostly proteins around 22 kDa, most possibly variants of the H1 histone family, with some proteins around 14 kDa belonging to the H2 family of histones. Type VIII-S sample proteins were mostly of 12-13 kDa (H4 histone family), with a few around 14 kDa from the H2 family of histones.

Samples were also analyzed after tryptic digestion. The untreated and PAD enzyme treated samples were subjected to proteolysis with a 10:1 enzyme to trypsin ratio. The buffer for proteolysis was MOPS pH 7.8 and the reaction was carried out for 20 min at 37°C. The proteolysis was stopped by adding formic acid to a final concentration of 5%. LC-MS/MS measurements were carried out using a Thermo Scientific Ultimate 3000 RSLC coupled to an LTQ-XL mass spectrometer. The sample components were separated on a 100x1 mm Hypersil GOLD aQ C18 column using mobile phases A – water with 0.15% formic acid and B – acetonitrile with 0.15% formic acid. The gradient elution started from 5% B kept for 1 minutes, increased to 44% B in 39 minutes with a flow rate of 0.085 mL/min. Column temperature was set to 30°C. Data was recorded in positive mode and Zoom scan settings in the 350-1400 m/z range, followed by 3 data dependent scans in the 200-2000 m/z range with a NCE of 45.

Raw LC-MS/MS data was converted to MGF format using MSConvert and database search was carried out using Waters ProteinLynx Global Server version 2.4 on latest Uniprot bovine proteome. Precursor mass tolerance was set to 0.35 Da and fragment tolerance to 0.6 Da. Enzyme specificity was required but 5 missed cleavages were allowed. For protein results 3 peptides were considered as the limit for acceptance. Database search also included histone specific modifications: acetylation, methylation and citrullination as variable modifications.

Results confirmed the sample compositions that was hinted at by the intact protein measurement. Main citrullination sites of the histones are collected in Table S1. Citrullination of H4 at residue 4 is only identified based on mass, as the peptide is too short for effective fragmentation. The amount of citrullination was found to be much higher in the VIII-S sample for the same citrullination sites. The extent of citrullination can be seen in the chromatograms included below. The chromatograms depicted in mauve show the citrullinated sample while the non citrullinated sample is shown in orange. When the citrullinated peptide is detected, a new peak appears in mauve. A decrease in orange peak height shows the uncitrullinated peptide containing sensitive arginine.

A

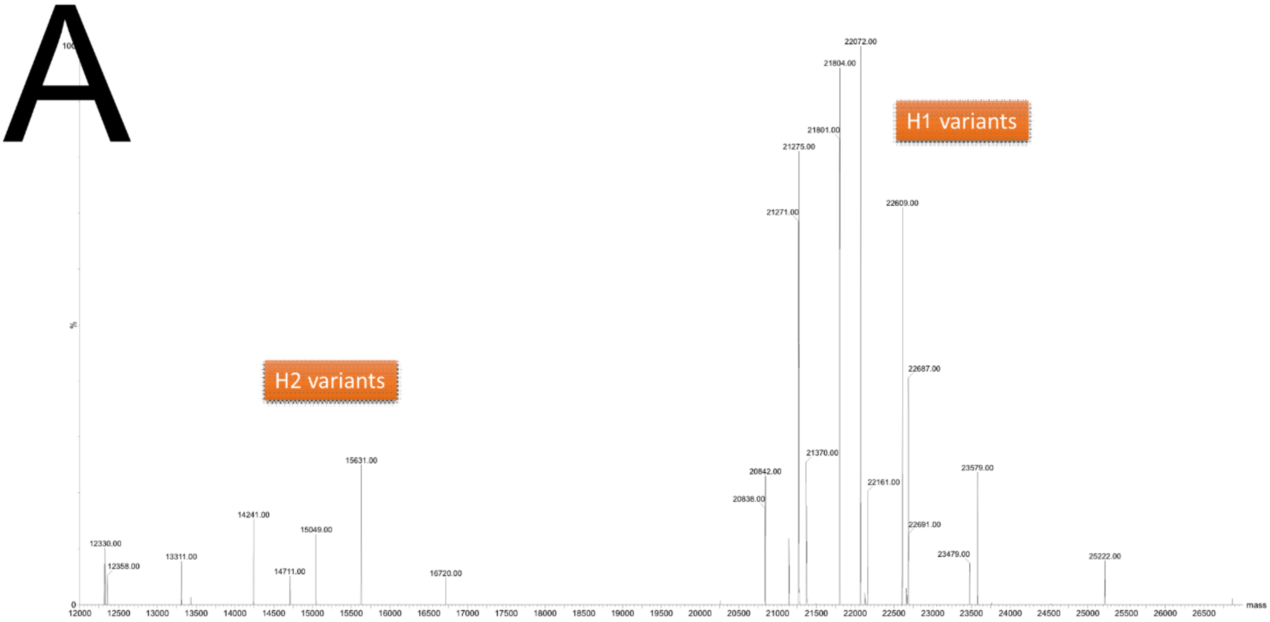

Accession	Entry	Description	mW (Da)	Peptides	Coverage (%)
A7MAZ5	H13_BOVIN	Histone H1 3 OS Bos taurus OX 9913 GN H1 3 PE 1 SV 1	22140	15	26.2443
G3N131	H11_BOVIN	Histone H1 1 OS Bos taurus OX 9913 GN H1 1 PE 1 SV 1	22087	11	25.6881
Q2M2T1	H2B1K_BOVIN	Histone H2B type 1 K OS Bos taurus OX 9913 GN H2BC12 PE 1 SV 3	13866	6	40.4762
P0C0S9	H2A1_BOVIN	Histone H2A type 1 OS Bos taurus OX 9913 PE 1 SV 2	14082	5	38.4615
A1A4R1	H2A2C_BOVIN	Histone H2A type 2 C OS Bos taurus OX 9913 GN H2AC20 PE 2 SV 1	13979	7	58.1395

B

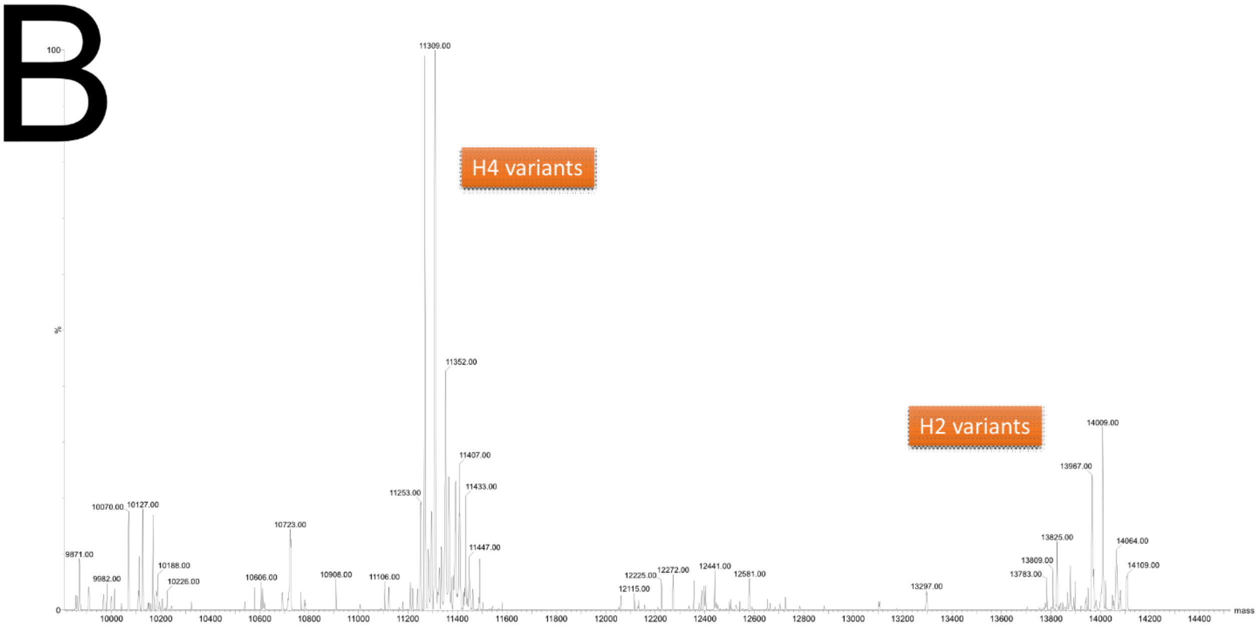

Accession	Entry	Description	mW (Da)	Peptides	Coverage (%)
A1A4R1	H2A2C_BOVIN	Histone H2A type 2 C OS Bos taurus OX 9913 GN H2AC20 PE 2 SV 1	13979	18	50.3876
P0C0S9	H2A1_BOVIN	Histone H2A type 1 OS Bos taurus OX 9913 PE 1 SV 2	14082	18	53.8462
P62803	H4_BOVIN	Histone H4 OS Bos taurus OX 9913 PE 1 SV 2	11360	17	82.5243
Q2M2T1	H2B1K_BOVIN	Histone H2B type 1 K OS Bos taurus OX 9913 GN H2BC12 PE 1 SV 3	13866	3	19.0476

Figure S1: Deconvoluted LC-MS spectra of and list of proteins identified in the bovine histone samples III-S (A) and VIII-S (B). The intact mass measurements show the components contribution to the sample composition.

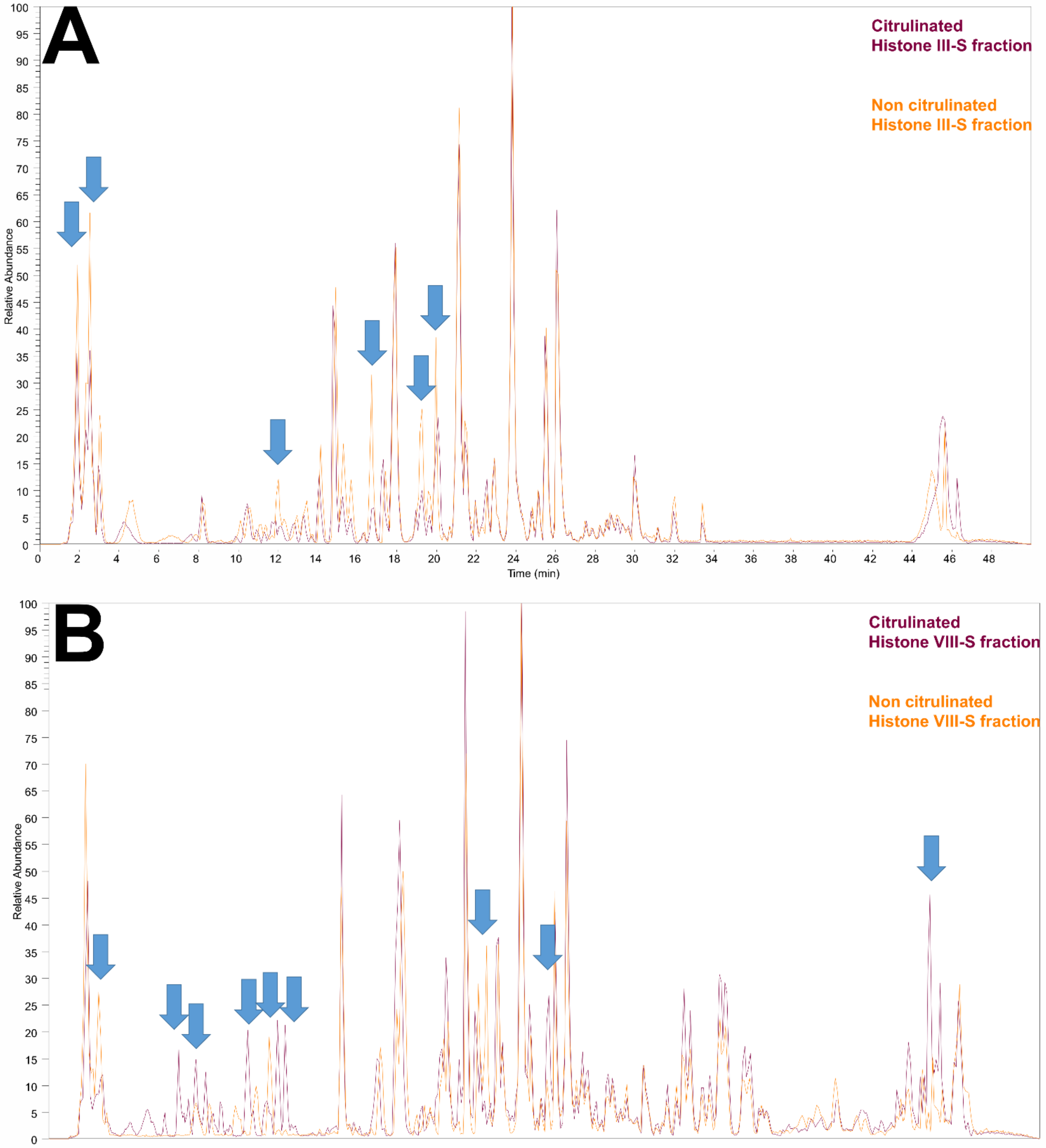

Figure S2: Comparison of citrullinated and uncitrullinated histone fractions for sample III-S (insert A) and sample VIII-S (insert B). The differemnces on the presented LC-MS/MS base peak chromatograms are due to PAD enzyme threatment. Arrows point to major changes in peak intensity. VIII-S sample shows more citrullination sites and with a higher extent, compared to III-S.

Table S1: Citrullination sites identified by MS/MS database search. Citrullination locations are reported including the leading methionine in the numbering

**Citrullinated Histone III-S**											
	H13_BOVIN	H11_BOVIN									
	m/z	Charge	Peak mW	Peptide mW	Delta (Da)	Ladder Score	Start	End	Sequence	Modifications	Site
	630.429	2	629.42	1258.72	0.12	76.81	54	65	(K)ERSGVSLAALKK(A)	Citrulline (2)	55
	853.9662	2	852.96	1706.86	0.07	68.82	65	80	(K)KALAAAGYDVEKNNSR(I)	Citrulline (16)	80
	H2B1K_BOVIN										
	m/z	Charge	Peak mW	Peptide mW	Delta (Da)	Ladder Score	Start	End	Sequence	Modifications	Site
	715.85547	2	714.85	1429.73	-0.04	64.00	74	86	(R)IAGEASRLAHYNK(R)	Citrulline (7)	80
	810.44885	2	809.44	1618.86	0.02	79.01	87	100	(K)RSTITSREIQTAVR(L)	Citrulline (1), Citrulline (7)	87,93
	731.8844	2	730.88	1461.78	-0.03	76.00	88	100	(R)STITSREIQTAVR(L)	Citrulline (6)	93
	H2A1_BOVIN	H2A2C_BOVIN									
	m/z	Charge	Peak mW	Peptide mW	Delta (Da)	Ladder Score	Start	End	Sequence	Modifications	Site
	767.4243	2	766.42	1532.81	0.03	71.60	17	30	(K)TRSSRAGLQFPVGR(V)	Citrulline (2), Citrulline (5)	18,21
	378.69598	2	377.69	755.47	-0.09	57.58	77	82	(K)TRIIPR(H)	Citrulline (2)	78

Citrullinated Histone VIII-S

	H2A2C_BOVIN	H2A1_BOVIN									
	m/z	Charge	Peak mW	Peptide mW	Delta (Da)	Ladder Score	Start	End	Sequence	Modifications	Site
	760.46295	2	759.46	1518.79	0.12	80.25	17.00	30	(K)SRSSRAGLQFPVGR(V)	Citrulline (2), Citrulline (5)	18, 21
	442.8142	2	441.81	883.56	0.05	79.49	76.00	82	(K)KTRIIPR(H)	Citrulline (3)	78
	665.9536	2	664.95	1329.82	0.07	69.84	79.00	89	(R)IIPRHLQLAIR(N)	Citrulline (4)	82
	H4_BOVIN										
	m/z	Charge	Peak mW	Peptide mW	Delta (Da)	Ladder Score	Start	End	Sequence	Modifications	Site
	847.96985	2	846.96	1693.98	-0.06	52.87	22.00	36	(K)VLRDNIQGITKPAIR(R)	Citrulline (3)	24
	1252.7726	2	1251.76	2503.29	0.24	38.52	81.00	103	(K)TVTAMDVVYALKRQGRTLYGFGG(-)	Citrulline (13)	93
